# Components of the Mediterranean Diet and Risk of COVID-19

**DOI:** 10.3389/fnut.2021.805533

**Published:** 2022-01-24

**Authors:** Rafael Perez-Araluce, Miguel Ángel Martínez-González, Alfredo Gea, Silvia Carlos

**Affiliations:** ^1^Department of Preventive Medicine and Public Health, University of Navarra, Pamplona, Spain; ^2^Centro de Investigación Biomédica en Red de Fisiopatología de la Obesidad y la Nutrición, Instituto de Salud Carlos III, Madrid, Spain; ^3^Navarra Institute for Health Research, Pamplona, Spain; ^4^Department of Nutrition, Harvard T.H. Chan School of Public Health, Harvard University, Boston, MA, United States

**Keywords:** Mediterranean diet, SARS-CoV-2, COVID-19, dairy products, SUN project

## Abstract

Adherence to the traditional Mediterranean diet has been customarily assessed with the Mediterranean diet score (MDS or Trichopolou Index), with values of 0 or 1 assigned to each of the nine elements, and with the use of the sex-specific median as the cutoff. The value of persons whose consumption of the six beneficial items (ratio of monounsaturated to saturated fatty acids, vegetables, legumes, fruits and nuts, cereal, and fish) is at or above the median and is assigned a value of 1. Otherwise they receive 0 points. For detrimental elements (meats and dairy products) persons whose consumption is below the median are assigned a value of 1. An additional ninth point is assigned to moderate ethanol intake. We assessed the effect of each of the nine components of the MDS (replacing the fats ratio with olive oil, the main source of monounsaturated fats in the Mediterranean diet) on the risk of COVID-19 infection, symptomatic and severe COVID-19. From March to December 2020, 9,699 participants of the “Seguimiento Universidad de Navarra” (SUN) cohort answered a COVID-19 questionnaire. After excluding doctors and nurses, 5,194 participants were included in the main statistical analyses. Among them, we observed 382 cases of COVID-19 based on symptoms and clinical diagnosis; 167 of them with test confirmation. For the two COVID-19 definitions used, we found a significant decrease in risk for a higher adherence to the Mediterranean diet (OR = 0.64, 95% CI: 0.42–0.98, *p* for trend = 0.040; and OR = 0.44, 95% CI: 0.22–0.88, *p* for trend = 0.020, for test-diagnosed cases). A protective effect was also found for symptomatic COVID-19 (OR = 0.64, 95% CI: 0.41–1.00, *p* for trend = 0.050). Among the different individual food groups, only the consumption of whole dairy products showed a harmful direct association. The Mediterranean diet as a whole seems more important than each of its components in preventing the infection and symptoms of COVID-19.

## Introduction

Since the beginning of the coronavirus disease-2019 (COVID-19) pandemic in December 2019 and by the end of October 2021 more than 245 million SARS-CoV-2 virus infections and near 5 million deaths had been notified (1).

It is now well-known that SARS-CoV-2 virus is mainly airborne-transmitted and thus, the most important preventive measures to avoid the inhalation of the virus are physical distancing, use of masks, and adequate ventilation (2). However, there are other non-hygiene related factors which can help reducing the risk of infection or its related clinical complications. As there are some comorbidities clearly associated with COVID-19, such as cardiovascular disease (CVD), type 2 diabetes, hypertension or obesity (3–7), in the long-term, previous adherence to a healthy lifestyle and diet could reduce the vulnerability of subjects to acquire the infection by reducing the burden of these chronic conditions. A key component of a healthy lifestyle is a balanced and healthy diet, which is in turn associated with an adequate immune response, as well as with antiinflammatory properties to reduce the risk of infections (8–13). Therefore, both the innate and adaptive immune cell functions against the entrance and replication of SARS-CoV-2 and the cytokine storm produced by this infection can be improved and modulated by the effect of an adequate nutritional status, in not-at-risk individuals as well as those with comorbidities (14). Additionally, diet is known to exert an impact on the human microbiota, which in turn can be associated with the risk of acquiring the virus or the development of symptoms after infection (15, 16).

The effect of nutrition on respiratory infections has been previously described (17, 18). Thus, early in June 2020 the World Health Organization Eastern Mediterranean Region Office published the “Nutrition advice for adults during the COVID-19 outbreak” supporting the relevance of nutrition on the pandemic (19). Additionally, since the very beginning of the pandemic, observational research and interventional studies focusing on nutritional aspects were carried out to find further non-pharmacological options and to prevent or reduce the health impacts of SARS-CoV-2 infection. Initially micronutrients were mainly analyzed, particularly vitamins (20, 21). Afterwards, the antioxidant, antiinflammatory, and antiviral activities of some nutrients and foods have been evaluated (6, 21–33). Less evidence is available for the effect of complete dietary patterns on COVID-19 (4, 32, 34–38), and most published reports on dietary patterns are related to the changes in diet caused by the pandemic or the lockdown.

In a previous assessment of the SUN (“Seguimiento Universidad de Navarra”) prospective cohort, our research group was pioneer in reporting that a better adherence to the Mediterranean diet was associated with a lower risk of COVID-19 among not health professionals (34). Thereafter, several studies conducted in other countries reported similar results (4, 32, 39). The effect that we previously reported assessed only the overall dietary pattern (not each specific component of the Mediterranean diet) and only the risk of acquiring the infection, not its severity. Thus, the objective of the present study is to analyze the effect of each of the components of the Mediterranean Diet and some other specific foods on COVID-19 infection and also on its severity.

## Materials and Methods

### Study Sample

The SUN Project is a prospective and multipurpose cohort study designed to evaluate different aspects of the Mediterranean dietary pattern and lifestyles, relating them to health outcomes. By the end of 2019 it already had nearly 23,000 participants, who are evaluated every 2 years through self-administered questionnaires. It is a dynamic cohort, and so, although the recruitment started in 1999, it is permanently open. Participants are university graduates from many different universities all over Spain. The methods and more specific details of the SUN cohort have already been described previously (40).

In addition to the biennial questionnaires, from March to December 2020, a specific questionnaire on COVID-19 was sent to all participants. In this questionnaire they were asked whether or not they had undergone a diagnostic test for COVID-19, they were also asked about a medical diagnosis of the disease and about different related symptoms. For all this information they were also asked to record the corresponding dates.

Given the high COVID-19 pandemic burden in Spain during 2020 and that the most relevant exposures for healthcare professionals are related to their work in clinical care and not so much to nutritional factors, our main analyses focused on those participants who were not healthcare professionals, although the analysis for the total number of participants was also included. This decision was based on consideration of the highly relevant differences in exposures between both types of participants in our cohort.

### Outcome Measurement

Two definitions to assess COVID-19 risk were used. One, more specific, included as incident cases only those participants who reported a positive diagnostic test. However, especially during the first months of the pandemic, there was a large under-diagnosis of the disease in Spain (41); consequently, not all cases were detected, potentially leaving a large number of false negatives. For a broader definition, participants with a medical diagnosis were also included as were those classified as incident cases by the Menni C. algorithm based on symptoms, age, and sex (42).

COVID-19 severity was also evaluated using two definitions. Those cases of COVID-19 that required hospitalization with symptoms compatible with the disease were classified as severe cases. Given the small number of participants with these characteristics, symptomatic cases were included in the severity analysis, and so symptomatic COVID-19 was included as a new outcome in our analysis. The symptoms included in this new definition were: cough, cold, respiratory distress, loss of smell or taste, diarrhea, and fever.

### Dietary Assessment

For dietary assessment, data were taken from a previously validated semiquantitative food frequency questionnaire (FFQ) (43). It consists of 136 items with consumption grouped in nine categories from “never or almost never” to “≥ 6 times a day.” From these data information on the consumption of different nutrients can also be obtained using data from updated Food Composition Tables (44).

This questionnaire is assessed at baseline and at 10 years of follow-up. Cumulative measures were used in those for whom information was available at both times.

The Mediterranean diet score (MDS) proposed by Trichopoulou was used to assess adherence to the Mediterranean diet. This index has been most widely used to assess adherence to the Mediterranean diet (45). The MDS considers nine components of the diet, 1 point corresponds to moderate ethanol intake (5–25 g/d for women and 10–50 g/d for men) and another point to the monounsaturated-to-saturated fatty acids ratio, the point is given to those with a ratio of monounsaturated to saturated fats at or above the sex-specific median. The other 7 points are for food groups: 1 point is given to those who have a consumption equal or higher than the sex-specific median consumption on each beneficial food groups (cereals, fruits and nuts, vegetables, legumes, and fish), or below the sex-specific median for two food groups that are not typical of the Mediterranean diet (meat and whole dairy products). The final punctuation ranges from 0 to 9.

Second, for evaluating each food separately, to minimize confounding by total energy intake, residuals based on total energy intake were calculated. As the ratio of monounsaturated to saturated fats did not correspond to a specific food, it was replaced with olive oil consumption. This approximation was adopted since olive oil was the main source of monounsaturated fatty acids in the traditional Mediterranean diet (45).

### Other Covariates

At baseline, standardized questionnaires were used for gathering information on demographic characteristics (age, sex, years of university education, occupation, and marital status), lifestyle habits (smoking status and physical activity) and anthropometric and clinical data (weight, height, and comorbidities).

This information was updated with different follow-up questionnaires. The diagnosis of new diseases and weight are updated in each of the follow-up questionnaires. Other variables, however, were updated less frequently, such as marital status (questionnaire after 14-year follow-up) or height (questionnaire after 10-year follow-up). For each of them the most recent available information was used.

### Statistical Analysis

First, a description of the different variables with different statistical parameters was carried out. For categorical variables, the percentage of participants included in each group was calculated, and for numerical variables the mean and standard deviation were calculated, or the median and the interquartile range were calculated if the distribution of the variable did not follow a normal distribution.

Secondly, to evaluate the possible effect of the adherence to the Mediterranean diet and its components on the incidence and severity of COVID-19, multivariable adjusted logistic regression models were used. The odds ratio (OR) for COVID-19 incidence was calculated according to both definitions established, symptomatic COVID-19 and severe COVID-19. For the Mediterranean diet a high adherence group (7 ≤ MDS ≤ 9) and an intermediate adherence group (4 ≤ MDS ≤ 6) were compared with a low adherence (MDS <4), used as reference.

For the different dietary components, the most extreme consumption categories were also compared. To do this, the energy-adjusted consumption of the different foods was divided into quintiles: the first quintile would correspond to the lowest consumption and the last to the highest consumption, with the other categories corresponding to intermediate levels of consumption.

The lowest consumption category was used as the reference category to evaluate foods typical of the Mediterranean diet (olive oil, fruits and nuts, vegetables, cereals, legumes, and fish). For foods that correspond to a negative score in the MDS, such as meat and whole dairy products, we used high consumption as the reference category. In addition, the effect of red meat and yogurt was also analyzed.

All ORs are expressed with their 95% confidence intervals. Participants below the percentile 1 or above the percentile 99 of total energy intake were excluded from the analysis.

All analyses were carried out both excluding and including healthcare professionals (HP), namely doctors and nurses. STATA program (version 16) was used for data analysis.

## Results

By mid-2021 the COVID-19 questionnaire of the SUN cohort had been answered by 9,699 participants. However, 73 of them did not coincide with a participant in the SUN Project database, so the final number of subjects initially included in this study was 9,626. After excluding participants with total energy intake beyond percentiles 1 and 99, the study sample included 9,485 participants.

Baseline characteristics of the population included in the study are shown in Table 1. It is a middle-aged population (mean age = 52.9 years), with a higher proportion of women, mostly married. Nearly one third of participants were doctors or nurses (32.5%), a very similar proportion of the total population of the SUN cohort. Regarding lifestyles, half of the participants had never smoked. A description of the most important conditions and chronic diseases potentially related to the risk of COVID-19 is also shown in Table 1.

**Table 1:** Baseline characteristics of the SUN cohort participants who completed the COVID-19 specific questionnaire.

	**All** **(*N* = 9,485)**	**Excluding HP** **(*N* = 6,406)**
**Demographic characteristics**		
Age (years), mean (SD)	52.9 (12.0)	52.8 (12.1)
Sex (female) (%)	62.3	59.1
Years of university education, median (IQR)	5 (4–5)	5 (4–5)
Marital status (%)		
Single	27.8	29.4
Married	63.3	62.5
Divorced	4.5	4.1
Others	4.4	4.0
Healthcare professionals (%)	32.5	–
**Lifestyle Habits**		
Smoking status (%)		
Current smoker	20.5	21.3
Former smoker	28.6	27.8
Physical activity (METS/week), median (IQR)	14 (5–27)	13 (5–27)
**Anthropometric and clinical data**		
Total energy intake (kcal/d), mean (SD)	2,438 (711)	2,436 (718)
Adherence to the MDS (0–9 score), mean (SD)	4.3 (1.8)	4.2 (1.8)
BMI (kg/m^2^), mean (SD)	24.3 (3.9)	24.3 (3.9)
Type 2-diabetes mellitus (%)	3.0	3.0
Hypertension (%)	21.0	21.3
Cardiovascular disease (%)	4.8	4.5
Cancer (%)	6.4	6.1
Pulmonary disease (%)	10.6	10.1

*HP, healthcare professionals; IQR, Interquartile Range; METS, Metabolic Equivalent Task; MDS, Mediterranean Diet Score; BMI, Body Mass Index*.

There were no significant differences in these characteristics when HP were excluded. However, we observed 373 (3.9%) cases of COVID-19 detected by a specific test for all our study population, but only 167 (2.6%) of them were among non-healthcare professionals. When we added those cases with clinical diagnosis and the ones predicted by the algorithm, the difference decreased: we had 666 (7%) for the total population and 382 (6%) for non-healthcare professionals. The percentages were even closer for the symptomatic cases, with 565 (6%) for all the study population and 338 (5.3%) when we excluded doctors and nurses. Finally, for serious cases, the percentages were the same, having 35 (0.4%) and 24 (0.4%) cases, respectively. When we assessed the interaction between adherence to the Mediterranean diet and being a healthcare professional, we found only a marginally significant *p* for interaction (*p* = 0.059) for test-diagnosed COVID-19 infection.

In Table 2, we describe the consumption of the different foods analyzed. We show the median consumption for each category after adding each participant's residual consumption to the average consumption.

**Table 2:** Median consumption of the foods analyzed across the different energy-adjusted quintiles of consumption of each food or food group.

**Food groups (g/day)**	**Low consumption (Q1)**	**Intermediate consumption (Q2–Q4)**	**High consumption (Q5)**
Olive oil	4	15	34
Fruits and nuts	79	297	640
Vegetables	139	323	605
Cereals	46	105	188
Legumes	10	21	35
Fish	41	90	163
Meat	98	173	261
Red meat	36	70	130
Whole dairy products	41	144	383
Yogurt	0	27	117

Our main analysis is shown in Table 3. There, we displayed the odds ratio of COVID-19 infection for non-healthcare professionals. For the two COVID-19 definitions, we found a significant decrease in risk with greater adherence to the Mediterranean diet. For the highest adherence category, we found a 56% decrease in risk (OR = 0.44, 95% CI: 0.22–0.88, *p* for trend = 0.020) when considering only cases with a positive test and 46% (OR = 0.64, 95% CI: 0.42–0.98, *p* for trend = 0.040) when using the broader definition. Among the different foods, only yogurt and the consumption of whole dairy products showed a significant direct association. In whole dairy products, the lowest consumption category had a 45% decreased risk (OR = 0.55, 95% CI: 0.33–0.94, *p* for trend = 0.027) and a 35% decrease in the risk of COVID-19 (OR = 0.65, 95% CI: 0.46–0.92, *p* for trend = 0.015), as measured in test-positive cases and in all possible cases, respectively. The results for yogurt are consistent with these results, showing an OR = 0.49 (0.28–0.86) and an OR = 0.71 (0.50–1.03) for the low consumption category, respectively, as compared with the highest consumption, although this last result was no longer significant (*p* for trend = 0.068).

**Table 3:** Multivariable adjusted* odds ratios (OR) and 95% confidence intervals (CI) of COVID-19 risk according to adherence to the Mediterranean diet (Mediterranean Diet Score, MDS) and consumption levels of different foods measured in residuals, excluding healthcare professionals.

		**COVID-19 (positive test)**			**COVID-19** ^‡^		
		***N* cases/*N* total**	**OR (and 95% CI)**	***p* for trend**	***N* cases/*N* total**	**OR (and 95% CI)**	***p* for trend**
Mediterranean diet	Low adherence	76/2,347	1 (Ref.)	0.020	158/2,347	1 (Ref.)	0.040
	Intermediate adherence	81/3,371	0.73 (0.52–1.01)		196/3,371	0.87 (0.70–1.09)	
	High adherence	10/688	0.44 (0.22–0.88)		28/688	0.64 (0.42–0.98)	
Olive oil	Low consumption (Q1)	31/1,310	1 (Ref.)	0.980	74/1,310	1 (Ref.)	0.738
	Intermediate consumption (Q2-Q4)	110/3,863	1.36 (0.90–2.08)		239/3,863	1.19 (0.90–1.57)	
	High consumption (Q5)	26/1,233	0.99 (0.58–1.71)		69/1,233	1.06 (0.75–1.51)	
Fruits and nuts	Low consumption (Q1)	43/1,336	1 (Ref.)	0.381	90/1,336	1 (Ref.)	0.962
	Intermediate consumption (Q2-Q4)	98/3,841	0.88 (0.59–1.31)		222/3,841	0.93 (0.70–1.22)	
	High consumption (Q5)	26/1,229	0.78 (0.45–1.35)		70/1,229	0.99 (0.69–1.42)	
Vegetables	Low consumption (Q1)	43/1,315	1 (Ref.)	0.963	84/1,315	1 (Ref.)	0.749
	Intermediate consumption (Q2-Q4)	92/3,886	0.80 (0.54–1.18)		228/3,886	1.01 (0.76–1.33)	
	High consumption (Q5)	32/1,205	1.01 (0.61–1.68)		70/1,205	1.06 (0.74–1.51)	
Cereals	Low consumption (Q1)	40/1,279	1 (Ref.)	0.268	79/1,279	1 (Ref.)	0.590
	Intermediate consumption (Q2-Q4)	99/3,848	0.83 (0.57–1.23)		237/3,848	1.06 (0.80–1.39)	
	High consumption (Q5)	28/1,279	0.75 (0.45–1.25)		66/1,279	0.91 (0.64–1.29)	
Legumes	Low consumption (Q1)	39/1,353	1 (Ref.)	0.987	86/1,353	1 (Ref.)	0.799
	Intermediate consumption (Q2-Q4)	95/3,816	0.90 (0.61–1.32)		223/3,816	0.95 (0.72–1.23)	
	High consumption (Q5)	33/1,237	1.00 (0.62–1.63)		73/1,237	0.96 (0.69–1.33)	
Fish	Low consumption (Q1)	42/1,348	1 (Ref.)	0.118	93/1,348	1 (Ref.)	0.062
	Intermediate consumption (Q2-Q4)	101/3,827	0.88 (0.60–1.29)		231/3,827	0.90 (0.69–1.17)	
	High consumption (Q5)	24/1,231	0.65 (0.38–1.12)		58/1,231	0.71 (0.50–1.02)	
Meat	High^***^ consumption	35/1,281	1 (Ref.)	0.323	87/1,281	1 (Ref.)	0.536
	Intermediate consumption (Q2-Q4)	109/3,855	1.14 (0.77–1.70)		225/3,855	0.90 (0.69–1.18)	
	Low consumption (Q1)	23/1,270	0.76 (0.43–1.32)		70/1,270	0.90 (0.64–1.26)	
Red meat^**^	High^***^ consumption	43/1,289	1 (Ref.)	0.415	81/1,289	1 (Ref.)	0.998
	Intermediate consumption (Q2-Q4)	93/3,845	0.76 (0.52–1.11)		226/3,845	0.96 (0.73–1.25)	
	Low consumption (Q1)	31/1,272	0.82 (0.50–1.33)		75/1,272	1.00 (0.71–1.40)	
Whole dairy products	High^***^ consumption	49/1,309	1 (Ref.)	0.027	104/1,309	1 (Ref.)	0.015
	Intermediate consumption (Q2-Q4)	94/3,868	0.67 (0.47–0.98)		213/3,868	0.67 (0.52–0.86)	
	Low consumption (Q1)	24/1,229	0.55 (0.33–0.94)		65/1,229	0.65 (0.46–0.92)	
Yogurt^**^	High^***^ consumption	42/1,289	1 (Ref.)	0.013	81/1,289	1 (Ref.)	0.068
	Intermediate consumption (Q2-Q4)	106/3,897	0.87 (0.60–1.26)		245/3,897	1.01 (0.77–1.31)	
	Low consumption (Q1)	19/1,220	0.49 (0.28–0.86)		56/1,220	0.71 (0.50–1.03)	
Alcohol^∨*^	Not moderate consumption	109/4,364	1 (Ref.)		267/4,364	1 (Ref.)	
	Moderate consumption	58/2,042	1.20 (0.86–1.68)	0.290	115/2,042	0.95 (0.75–1.20)	0.641

*Adjusted for sex, age, years of university studies, marital status, smoking, BMI, physical activity at leisure-time, year of entering the cohort, previous diagnosis of chronic diseases (diabetes, hypertension, cardiovascular disease, cancer or pulmonary disease) and category of consumption of the other food groups.

** *In the adjustment for the calculation of the ORs of red meat and yogurt, the total consumption of meat and whole dairy products, respectively, had not been included in the model*.

*** *For foods that are not typical of the Mediterranean diet (meat, red meat, whole dairy products and yogurt), the reference category was changed to the highest consumption category*.

∨* *With respect to alcohol, moderate vs. non-moderate consumption was compared. Therefore, the p-value is not a p for trend*.

‡ *This definition of COVID-19 includes participants with a positive test and medical diagnosis, as well as those classified as incident cases by the Menni C. algorithm based on symptoms, age, and sex*.

For other foods, such as fish, although a point estimate for the OR was lower than the null value and a certain linear trend was seen, the confidence intervals were wide and they included the null value and consequently, the *p* for trend was not significant.

When we included healthcare professionals (see Table 4), the Mediterranean diet continued to have OR values below 1 for the point estimate, and with a linear trend, but the results were not significant. For COVID-19 cases with positive test, the only significant result was olive oil, with an OR = 1.53 (1.07–2.18) for the high consumption category. The adverse association for meat consumption approached significance and achieved it when we extended the COVID-19 cases to all possible. This result would show a lower risk of COVID-19 for lower meat consumption [OR = 0.76 (0.58–0.99)]. With this definition the result for whole dairy product was also close to being significant.

**Table 4:** Multivariable adjusted* odds ratios (OR) and 95% confidence intervals (CI) of COVID-19 risk according to adherence to the Mediterranean diet (Mediterranean Diet Score, MDS) and consumption levels of different foods measured in residuals, including healthcare professionals.

		**COVID-19 (positive test)**			**COVID-19** ^‡^		
		***N* cases/*N* total**	**OR (and 95% CI)**	***p* for trend**	***N* cases/*N* total**	**OR (and 95% CI)**	***p* for trend**
Mediterranean diet	Low adherence	142/3,300	1 (Ref.)	0.166	248/3,300	1 (Ref.)	0.137
	Intermediate adherence	199/5,082	0.94 (0.75–1.18)		360/5,082	0.98 (0.83–1.17)	
	High adherence	32/1,103	0.75 (0.49–1.13)		58/1,103	0.79 (0.58–1.08)	
Olive oil	Low consumption (Q1)	58/1,898	1 (Ref.)	0.020	118/1,898	1 (Ref.)	0.099
	Intermediate consumption (Q2-Q4)	234/5,692	1.47 (1.09–1.99)		412/5,692	1.26 (1.01–1.56)	
	High consumption (Q5)	81/1,895	1.53 (1.07–2.18)		136/1,895	1.25 (0.96–1.63)	
Fruits and nuts	Low consumption (Q1)	78/1,899	1 (Ref.)	0.108	142/1,899	1 (Ref.)	0.250
	Intermediate consumption (Q2-Q4)	243/5,690	1.14 (0.86–1.50)		409/5,690	1.04 (0.84–1.29)	
	High consumption (Q5)	52/1,896	0.76 (0.52–1.12)		115/1,896	0.94 (0.71–1.25)	
Vegetables	Low consumption (Q1)	72/1,898	1 (Ref.)	0.166	128/1,898	1 (Ref.)	0.684
	Intermediate consumption (Q2-Q4)	230/5,691	1.13 (0.84–1.51)		410/5,691	1.15 (0.93–1.44)	
	High consumption (Q5)	71/1,896	1.11 (0.77–1.60)		128/1,896	1.13 (0.86–1.49)	
Cereals	Low consumption (Q1)	86/1,898	1 (Ref.)	0.559	147/1,898	1 (Ref.)	0.371
	Intermediate consumption (Q2-Q4)	224/5,691	0.83 (0.64–1.08)		400/5,691	0.91 (0.74–1.11)	
	High consumption (Q5)	63/1,896	0.75 (0.53–1.06)		119/1,896	0.86 (0.66–1.11)	
Legumes	Low consumption (Q1)	76/1,898	1 (Ref.)	0.683	143/1,898	1 (Ref.)	0.600
	Intermediate consumption (Q2-Q4)	215/5,691	0.91 (0.69–1.20)		387/5,691	0.88 (0.72–1.09)	
	High consumption (Q5)	82/1,896	1.07 (0.77–1.49)		136/1,896	0.93 (0.73–1.20)	
Fish	Low consumption (Q1)	92/1,898	1 (Ref.)	0.108	156/1,898	1 (Ref.)	0.144
	Intermediate consumption (Q2-Q4)	208/5,691	0.68 (0.52–0.89)		383/5,691	0.79 (0.64–0.97)	
	High consumption (Q5)	73/1,896	0.76 (0.54–1.06)		127/1,896	0.82 (0.63–1.07)	
Meat	High^***^ consumption	82/1,896	1 (Ref.)	0.057	153/1,896	1 (Ref.)	0.045
	Intermediate consumption (Q2-Q4)	243/5,691	1.09 (0.84–1.43)		410/5,691	0.96 (0.78–1.17)	
	Low consumption (Q1)	48/1,898	0.69 (0.47–1.01)		103/1,898	0.76 (0.58–0.99)	
Red meat^**^	High^***^ consumption	91/1,896	1 (Ref.)	0.268	91/1,896	1 (Ref.)	0.392
	Intermediate consumption (Q2-Q4)	211/5,691	0.78 (0.60–1.01)		211/5,691	0.97 (0.79–1.18)	
	Low consumption (Q1)	71/1,898	0.83 (0.60–1.15)		71/1,898	0.89 (0.69–1.16)	
Whole dairy products	High^***^ consumption	84/1,896	1 (Ref.)	0.386	154/1,896	1 (Ref.)	0.059
	Intermediate consumption (Q2-Q4)	223/5,691	0.89 (0.68–1.17)		395/5,691	0.84 (0.68–1.03)	
	Low consumption (Q1)	66/1,898	0.86 (0.60–1.22)		117/1,898	0.77 (0.59–1.01)	
Yogurt^**^	High^***^ consumption	83/1,896	1 (Ref.)	0.307	83/1,896	1 (Ref.)	0.152
	Intermediate consumption (Q2-Q4)	226/5,691	0.93 (0.71–1.21)		226/5,691	1.04 (0.85–1.28)	
	Low consumption (Q1)	64/1,898	0.83 (0.59–1.18)		64/1,898	0.82 (0.63–1.08)	
Alcohol^∨*^	Not moderate consumption	259/6,542	1 (Ref.)		472/6,542	1 (Ref.)	
	Moderate consumption	114/2,943	1.13 (0.89–1.43)	0.305	194/2,943	0.99 (0.83–1.19)	0.937

*Adjusted for sex, age, years of university studies, marital status, smoking, BMI, physical activity at leisure-time, year of entering the cohort, previous diagnosis of chronic diseases (diabetes, hypertension, cardiovascular disease, cancer or pulmonary disease) and category of consumption of the other food groups.

** *In the adjustment for the calculation of the ORs of red meat and yogurt, the total consumption of meat and whole dairy products, respectively, had not been included in the model*.

*** *For foods that are not typical of the Mediterranean diet (meat, red meat, whole dairy products and yogurt), the reference category was changed to the highest consumption category*.

∨* *With respect to alcohol, moderate vs. non-moderate consumption was compared. Therefore, the p-value is not a p for trend*.

‡ *This definition of COVID-19 includes participants with a positive test and medical diagnosis, as well as those classified as incident cases by the Menni C. algorithm based on symptoms, age, and sex*.

In Tables 5, 6, we evaluated the relationship between the Mediterranean diet and each of its particular components with the risk of symptomatic and severe COVID-19, both excluding and including healthcare professionals respectively.

**Table 5:** Multivariable adjusted* odds ratios (OR) and 95% confidence intervals (CI) of symptomatic and severe COVID-19 risk according to adherence to the Mediterranean diet (Mediterranean Diet Score, MDS) and consumption levels of different foods measured in residuals, excluding healthcare professionals.

		**Symptomatic COVID-19**			**Serious COVID-19**		
		***N* cases/*N* total**	**OR (and 95% CI)**	***p* for trend**	***N* cases/*N* total**	**OR (and 95% CI)**	***p* for trend**
Mediterranean diet	Low adherence	143/2,347	1 (Ref.)	0.050	7/2,347	1 (Ref.)	0.270
	Intermediate adherence	169/3,371	0.82 (0.65–1.04)		16/3,371	1.28 (0.51–3.24)	
	High adherence	26/688	0.64 (0.41–1.00)		1/688	0.30 (0.03–2.57)	
Olive oil	Low consumption (Q1)	70/1,310	1 (Ref.)	0.714	7/1,310	1 (Ref.)	0.619
	Intermediate consumption (Q2-Q4)	210/3,863	1.10 (0.82–1.47)		12/3,863	0.57 (0.21–1.55)	
	High consumption (Q5)	58/1,233	0.93 (0.65–1.35)		5/1,233	0.74 (0.22–2.45)	
Fruits and nuts	Low consumption (Q1)	81/1,336	1 (Ref.)	0.798	5/1,336	1 (Ref.)	0.090
	Intermediate consumption (Q2-Q4)	192/3,841	0.90 (0.68–1.21)		12/3,841	1.30 (0.42–4.03)	
	High consumption (Q5)	65/1,229	1.05 (0.72–1.52)		7/1,229	3.17 (0.83–12.08)	
Vegetables	Low consumption (Q1)	75/1,315	1 (Ref.)	0.519	8/1,315	1 (Ref.)	0.144
	Intermediate consumption (Q2-Q4)	209/3,886	1.05 (0.78–1.40)		13/3,886	0.52 (0.20–1.37)	
	High consumption (Q5)	54/1,205	0.88 (0.60–1.30)		3/1,205	0.34 (0.08–1.45)	
Cereals	Low consumption (Q1)	73/1,279	1 (Ref.)	0.534	5/1,279	1 (Ref.)	0.627
	Intermediate consumption (Q2-Q4)	205/3,848	0.99 (0.74–1.32)		13/3,848	1.08 (0.36–3.19)	
	High consumption (Q5)	60/1,279	0.89 (0.62–1.28)		6/1,279	1.37 (0.38–4.91)	
Legumes	Low consumption (Q1)	78/1,353	1 (Ref.)	0.470	11/1,353	1 (Ref.)	0.047
	Intermediate consumption (Q2-Q4)	199/3,816	0.94 (0.71–1.24)		11/3,816	0.37 (0.15–0.90)	
	High consumption (Q5)	61/1,237	0.88 (0.62–1.25)		2/1,237	0.21 (0.04–0.98)	
Fish	Low consumption (Q1)	82/1,348	1 (Ref.)	0.155	6/1,348	1 (Ref.)	0.939
	Intermediate consumption (Q2-Q4)	203/3,827	0.92 (0.69–1.21)		14/3,827	0.99 (0.36–2.73)	
	High consumption (Q5)	53/1,231	0.76 (0.52–1.11)		4/1,231	0.95 (0.24–3.76)	
Meat	High^***^ consumption	82/1,281	1 (Ref.)	0.563	5/1,281	1 (Ref.)	0.628
	Intermediate consumption (Q2-Q4)	191/3,855	0.82 (0.62–1.08)		14/3,855	0.96 (0.33–2.83)	
	Low consumption (Q1)	65/1,270	0.90 (0.63–1.28)		5/1,270	0.72 (0.19–2.74)	
Red meat^**^	High^***^ consumption	68/1,289	1 (Ref.)	0.744	5/1,289	1 (Ref.)	0.721
	Intermediate consumption (Q2-Q4)	204/3,845	1.04 (0.78–1.39)		14/3,845	0.98 (0.34–2.84)	
	Low consumption (Q1)	66/1,272	1.06 (0.74–1.53)		5/1,272	0.79 (0.21–2.94)	
Whole dairy products	High^***^ consumption	89/1,309	1 (Ref.)	0.049	7/1,309	1 (Ref.)	0.190
	Intermediate consumption (Q2-Q4)	190/3,868	0.71 (0.54–0.93)		13/3,868	0.59 (0.22–1.57)	
	Low consumption (Q1)	59/1,229	0.69 (0.48–1.00)		4/1,229	0.41 (0.11–1.56)	
Yogurt^**^	High^***^ consumption	68/1,289	1 (Ref.)	0.307	6/1,289	1 (Ref.)	0.197
	Intermediate consumption (Q2-Q4)	216/3,897	1.07 (0.80–1.43)		15/3,897	0.86 (0.32–2.34)	
	Low consumption (Q1)	54/1,220	0.82 (0.56–1.20)		3/1,220	0.38 (0.09–1.64)	
Alcohol^∨*^	Not moderate consumption	235/4,364	1 (Ref.)		15/4,364	1 (Ref.)	
	Moderate consumption	103/2,042	0.96 (0.75–1.23)	0.737	58/2,042	1.17 (0.49–2.80)	0.716

*Adjusted for sex, age, years of university studies, marital status, smoking, BMI, physical activity at leisure-time, year of entering the cohort, previous diagnosis of chronic diseases (diabetes, hypertension, cardiovascular disease, cancer or pulmonary disease) and category of consumption of the other food groups.

** *In the adjustment for the calculation of the ORs of red meat and yogurt, the total consumption of meat and whole dairy products, respectively, had not been included in the model*.

*** *For foods that are not typical of the Mediterranean diet (meat, red meat, whole dairy products and yogurt), the reference category was changed to the highest consumption category*.

∨* *With respect to alcohol, moderate vs. non-moderate consumption was compared. Therefore, the p-value is not a p for trend*.

**Table 6:** Multivariable adjusted* Odds ratios (OR) and 95% confidence intervals (CI) of symptomatic and severe COVID-19 risk according to adherence to the Mediterranean diet (Mediterranean Diet Score, MDS) and consumption levels of different foods measured in residuals, including healthcare professionals.

		**Symptomatic COVID-19**			**Serious COVID-19**		
		***N* cases/*N* total**	**OR (and 95% CI)**	***p* for trend**	***N* cases/*N* total**	**OR (and 95% CI)**	***p* for trend**
Mediterranean diet	Low adherence	214/3,300	1 (Ref.)	0.294	10/3,300	1 (Ref.)	0.153
	Intermediate adherence	299/5,082	0.95 (0.79–1.15)		24/5,082	1.39 (0.64–3.00)	
	High adherence	52/1,103	0.84 (0.60–1.16)		1/1,103	0.22 (0.03–1.77)	
Olive oil	Low consumption (Q1)	100/1,898	1 (Ref.)	0.274	9/1,898	1 (Ref.)	0.626
	Intermediate consumption (Q2-Q4)	355/5,692	1.26 (0.99–1.60)		19/5,692	0.69 (0.30–1.60)	
	High consumption (Q5)	110/1,895	1.17 (0.88–1.57)		7/1,895	0.77 (0.28–2.17)	
Fruits and nuts	Low consumption (Q1)	119/1,899	1 (Ref.)	0.294	7/1,899	1 (Ref.)	0.249
	Intermediate consumption (Q2-Q4)	345/5,690	1.04 (0.83–1.31)		20/5,690	1.15 (0.46–2.90)	
	High consumption (Q5)	101/1,896	0.99 (0.73–1.33)		8/1,896	1.70 (0.55–5.28)	
Vegetables	Low consumption (Q1)	110/1,898	1 (Ref.)	0.941	9/1,898	1 (Ref.)	0.361
	Intermediate consumption (Q2-Q4)	354/5,691	1.13 (0.90–1.44)		21/5,691	0.81 (0.35–1.89)	
	High consumption (Q5)	101/1,896	1.00 (0.74–1.35)		5/1,896	0.56 (0.17–1.86)	
Cereals	Low consumption (Q1)	124/1,898	1 (Ref.)	0.991	6/1,898	1 (Ref.)	0.344
	Intermediate consumption (Q2-Q4)	339/5,691	0.90 (0.72–1.12)		19/5,691	1.19 (0.46–3.08)	
	High consumption (Q5)	102/1,896	0.86 (0.65–1.14)		10/1,896	1.87 (0.64–5.43)	
Legumes	Low consumption (Q1)	119/1,898	1 (Ref.)	0.509	12/1,898	1 (Ref.)	0.056
	Intermediate consumption (Q2-Q4)	335/5,691	0.92 (0.74–1.15)		19/5,691	0.50 (0.23–1.07)	
	High consumption (Q5)	111/1,896	0.91 (0.69–1.20)		4/1,896	0.32 (0.10–1.03)	
Fish	Low consumption (Q1)	128/1,898	1 (Ref.)	0.497	6/1,898	1 (Ref.)	0.310
	Intermediate consumption (Q2-Q4)	326/5,691	0.83 (0.67–1.04)		21/5,691	1.35 (0.52–3.47)	
	High consumption (Q5)	111/1,896	0.91 (0.68–1.20)		8/1,896	1.81 (0.58–5.68)	
Meat	Hig^***^ consumption	126/1,896	1 (Ref.)	0.190	6/1,896	1 (Ref.)	0.941
	Intermediate consumption (Q2-Q4)	348/5,691	0.99 (0.80–1.23)		22/5,691	1.15 (0.45–2.95)	
	Low consumption (Q1)	91/1,898	0.82 (0.61–1.10)		7/1,898	0.96 (0.30–3.05)	
Red meat^**^	Hig^***^ consumption	91/1,896	1 (Ref.)	0.569	91/1,896	1 (Ref.)	0.943
	Intermediate consumption (Q2-Q4)	211/5,691	1.07 (0.86–1.34)		211/5,691	1.19 (0.47–2.99)	
	Low consumption (Q1)	71/1,898	0.92 (0.69–1.23)		71/1,898	1.04 (0.34–3.24)	
Whole dairy products	Hig^***^ consumption	125/1,896	1 (Ref.)	0.282	10/1,896	1 (Ref.)	0.090
	Intermediate consumption (Q2-Q4)	338/5,691	0.89 (0.71–1.11)		20/5,691	0.60 (0.27–1.35)	
	Low consumption (Q1)	102/1,898	0.85 (0.64–1.14)		5/1,898	0.37 (0.12–1.17)	
Yogurt^**^	Hig^***^ consumption	83/1,896	1 (Ref.)	0.789	83/1,896	1 (Ref.)	0.052
	Intermediate consumption (Q2-Q4)	226/5,691	1.17 (0.93–1.47)		226/5,691	0.66 (0.30–1.44)	
	Low consumption (Q1)	64/1,898	0.96 (0.72–1.29)		64/1,898	0.30 (0.09–1.01)	
Alcohol^∨*^	Not moderate consumption	396/6,542	1 (Ref.)		22/6,542	1 (Ref.)	
	Moderate consumption	169/2,943	1.02 (0.84–1.24)	0.819	13/2,943	1.19 (0.58–2.43)	0.627

*Adjusted for sex, age, years of university studies, marital status, smoking, BMI, physical activity at leisure-time, year of entering the cohort, previous diagnosis of chronic diseases (diabetes, hypertension, cardiovascular disease, cancer or pulmonary disease) and category of consumption of the other food groups.

** *In the adjustment for the calculation of the ORs of red meat and yogurt, the total consumption of meat and whole dairy products, respectively, had not been included in the model*.

*** *For foods that are not typical of the Mediterranean diet (meat, red meat, whole dairy products and yogurt), the reference category was changed to the highest consumption category*.

∨* *With respect to alcohol, moderate vs. non-moderate consumption was compared. Therefore, the p-value is not a p for trend*.

When we excluded healthcare professionals, the inverse association of the Mediterranean diet with the risk of symptomatic COVID-19 showed a *p* for trend = 0.050. The effect of high adherence showed a protection of 66% [OR = 0.64 (0.41–1.00)] at the limit of statistical significance. Regarding its particular components, the only one with a detrimental association was again a low consumption of whole dairy products [OR = 0.69 (0.48–1.00) for low vs. high consumption]. For serious COVID-19 cases, the inverse protective association with a high consumption of legumes deserves to be emphasized [OR = 0.21 (0.04–0.98) for high vs. low consumption].

In Table 6, also adding health professionals we mainly found non-significant results for severe or symptomatic COVID-19.

## Discussion

As previously published by our research group (34), the Mediterranean Diet (MedDiet) showed a significant protective effect against COVID-19. In this work, we have further analyzed the effect of MedDiet on COVID-19, considering new definitions of the disease, as well as including severity as an outcome. This dietary pattern remained protective when apart from a COVID-19 positive test, symptoms, and medical-diagnosis were also included, and a linear trend was observed. For severe COVID-19 the incidence was very low, so no significant results were found (there was only one case of severe COVID-19 among participants with high MedDiet adherence).

Mediterranean Diet has been mainly characterized by having antiinflammatory properties, intestinal microbiota modulation, and immune protective effect, which may protect against COVID-19 (16, 23), in addition, its benefits have been attributed to these characteristics also for many non-communicable diseases (46). However, when the effect on COVID-19 incidence has been analyzed for individual foods, overall, significant associations have been scarcely found.

Among the different food groups included in the MDS defined by Trichopoulou (45), the only result that can be highlighted is the detrimental effect of whole dairy products. A significant linear association was found between a low consumption of whole dairy products and lower risk of COVID-19 positive test as well as symptomatic COVID-19. Likewise, consumption of yogurt was directly associated with the risk of having a positive COVID-19 test, and a linear trend was found for this association.

There is scarce evidence on these associations. The effect of dairy on COVID-19 is greatly focused on fermented dairy products, mainly on those including probiotics, which is not consistent with our results, and are associated with an enhanced immunity and lower risk of infectious diseases, including COVID-19 (mainly based on the modulation of intestinal microbiome) (15, 16, 30, 35, 47, 48). Similarly, there is evidence on the immune modulatory role of raw cow's milk on respiratory infections (49), and even a recent publication has also shown activity against SARS-CoV-2 of some milk-derived bioactive peptides (47, 50). However, there are also publications that describe mechanisms that could explain the association found in this work. For instance, some authors have described the possibility of having acquired SARS-CoV-2 through contamination by dairy products (51, 52).

Another study, although of ecological design, which analyzed the correlation between different foods and nutrients and COVID-19 infection and mortality worldwide, found that countries with higher calcium intake had higher infection rates (29). The authors also found an increased crude infection rate for increased consumption of milk. Spain is a country with a high incidence of COVID-19 and is also among the countries with the highest milk consumption worldwide (53), so this possible ecological explanation could be further analyzed.

Another related hypothesis, also recently described by Deschasaux-Tanguy et al. (54), could be due to the calcium content of dairy products. Different studies have shown a relation between calcium levels and COVID-19 (55, 56), although the mechanism of the causal relation is not clear yet. For other viruses, including SARS-CoV and MERS-CoV, calcium plays an important role in their infectiousness and replication (57–59). Likewise, a low consumption of dairy products and consequently, lower calcium levels could limit the entrance and replication of SARS-CoV-2. Also, a protective effect of vitamin D against SARS-CoV-2 has been described as a result of its immunomodulatory properties on macrophages, monocytes, dendritic cells, and T and B lymphocytes (60, 61). As there is low consistent evidence on the association between dairy products and COVID-19, more plausible explanations, biological and nutritional mechanisms should be explored to explain this association. Apart from yogurt, the specific effect of other dairy products which have not been analyzed in this work should be studied in more detail; this could help understand the observed effect for dairies.

With respect to other food groups, for legumes, we found a significant protective effect, only for COVID-19 severity. However, as noted above, it is important to consider the low number of cases of severe COVID-19.

For the rest of the components, no effect was observed. For olive oil, despite its antioxidant and anti-inflammatory effect, no individual effect was seen. This could be partly explained by the fact that we do not collect the different types of olive oil and these effects are mainly found in extra virgin olive oil. This type of oil, which has a higher unsaponifiable fraction where the components (vitamins, polyphenols) that give extra virgin olive oil these properties, are found (33). The same unexpected results as for olive oil were found for vegetables and fruit and nuts. Our results are not consistent with those found by Vu and cols. who analyzed in the UK Biobank the association of different food groups and incident COVID-19. They showed that a higher consumption of vegetables was independently associated with lower odds of COVID-19, which could be explained by their antiinflammatory and antiviral properties (27). On the other hand, there are also studies that have shown a relationship between higher fruit consumption and SARS-CoV-2 infection (29).

Fish and cereals have protective ORs and “a certain linear trend”; however, their confidence intervals include the null value, and so the final results were not significant. Other authors also show no significant association for fish consumption and COVID-19 (27). Perhaps an effect might be expected because of their vitamin D or omega-3 fatty acid content, which has shown some effect on COVID-19 as a result of their impact on the expression of pro- and antiinflammatory cytokines (10, 12, 21, 31, 62–64). In this work, whether it was oily fish or not was not evaluated. Regarding cereals, an antiinflammatory effect related to their fiber fermentation by the gut microbiota has been described (23).

It should be noted that our results are focused on non-healthcare participants. It has been considered that the general characteristics of this population were similar to those of non-healthcare workers and that healthcare personnel were at additional risk of COVID-19, especially at the time of the study, and that priority was given in this group to diagnostic testing (65, 66). This approach has been taken into account to overcome one of our major limitations, which is not having been able to measure several other exposures of the participants, according to the rigor of compliance with non-pharmacological preventive measures (masks, physical distancing, hand washing, and ventilation).

Finally, we can conclude that the effect of the complete Mediterranean dietary pattern is more important than that of its individual food groups. On one hand, for some foods, there was not a big difference between the most extreme consumption categories; therefore, the effect of high or low consumption would be more difficult to be observed. As more foods are included, these differences will grow. On the other hand, although we found a significant inverse association between dairy products and COVID-19, the effect of the Mediterranean diet is the combined effect of all its components, which can combine and enhance each other. Undoubtedly, the recommendation of a complete pattern is much more effective than that of isolated foods.

## Data Availability Statement

The original contributions presented in the study are included in the article/supplementary material, further inquiries can be directed to the corresponding author/s.

## Ethics Statement

The studies involving human participants were reviewed and approved by the Research Ethics Committee of the University of Navarra. The patients/participants provided their written informed consent to participate in this study.

## Author Contributions

RP-A, MM-G, and SC participated in the design of the study. RP-A was involved in the data analysis, supervised by SC and MM-G. SC and RP-A participated in writing the original manuscript. Finally, the manuscript was reviewed by MM-G and AG. All authors contributed to the article and approved the submitted version.

## Funding

The SUN Project has received funding from the Spanish Government-Instituto de Salud Carlos III, and the European Regional Development Fund (FEDER) (RD 06/0045, CIBER-OBN, Grants PI10/02658, PI10/02293, PI13/00615, PI14/01668, PI14/01798, PI14/01764, PI17/01795, and G03/140), the Navarra Regional Government (27/2011, 45/2011, 122/2014), the Government Delegation for the National Drug Plan (2020/ 021), and the University of Navarra.

## Conflict of Interest

The authors declare that the research was conducted in the absence of any commercial or financial relationships that could be construed as a potential conflict of interest.

## Publisher's Note

All claims expressed in this article are solely those of the authors and do not necessarily represent those of their affiliated organizations, or those of the publisher, the editors and the reviewers. Any product that may be evaluated in this article, or claim that may be made by its manufacturer, is not guaranteed or endorsed by the publisher.
